# The influence of occupational heat stress on serum inflammatory cytokines among traditional bakery workers in Iran

**DOI:** 10.1371/journal.pone.0302847

**Published:** 2024-05-06

**Authors:** Zahra Mirsanei, Yahya Asemani, Milad Derakhshanjazari, Vahid Gharibi, Pirasteh Norouzi, Sepideh Mahdavi, Rosanna Cousins

**Affiliations:** 1 Department of Immunology, Shahid Beheshti University of Medical Sciences, Tehran, Iran; 2 Department of Immunology, Student Research Committee, Shahid Beheshti University of Medical Sciences, Tehran, Iran; 3 Department of Occupational Health, Neyshabur University of Medical Sciences, Neyshabur, Iran; 4 Department of Occupational Health and Safety Engineering, School of Public Health, Arak University of Medical Sciences, Arak, Iran; 5 Department of Physiology, School of Medicine, Shahroud University of Medical Sciences, Shahroud, Iran; 6 Department of Epidemiology, School of Public Health, Iran University of Medical Sciences, Tehran, Iran; 7 Department of Psychology, Liverpool Hope University, Liverpool, United Kingdom; Drexel University School of Public Health, UNITED STATES

## Abstract

Heat exposure exceeding the ISO7243:1989 standard limit can contribute to health problems among employees in a variety of workplaces. Ignoring heat standard requirements in hot working conditions such as bakeries results in physiologic and health problems, as well as an elevated risk of later illnesses. In this analytical case-control study, the serum levels of four inflammatory factors (interleukin-1 beta, interleukin-6, tumor necrosis factor-α, and C-reactive protein) were assessed using an enzyme-linked immunosorbent assay. 105 male artisan bakers (in four job classifications in bakeries and staff) were compared based on demographic characteristics and inflammatory factors. The findings of the study showed correlations between serum interleukin-1β, interleukin-6, and C-reactive protein levels and thermal exposure in the occupational environment and employment type. Moreover, some differences in serum level of interleukin-1β and job type were observed. Heat overexposure affected the increase of interleukin-1β and C-reactive protein secretion. As a result of years of working in high-temperature conditions, inflammation can lead to subsequent diseases in workers. To protect their health from this occupational hazard, additional safeguards are needed. Our recommendations could also be applied to overly hot work environments that may cause heat stress in workers.

## Introduction

Heat exposure has been identified as a health risk factor, and one of the most serious health hazards among people who work in hot conditions [1,2]. Humans, as warm-blooded creatures, can only survive and execute social and professional activities within a specific temperature range [3,4]. Most people are comfortable when the temperature is 21–24°C and the relative humidity is 50–60% [5,6]. Various animal and human studies have examined the effects of increased ambient temperatures–either as a result of climate change or as a result of employment conditions–on the body’s health and physiology, including the cardiovascular [7], respiratory [8], endocrine [9], urinary [10], proteomic [11], and reproductive systems [12].

Studies show that due to climate change and global warming, heat stress incidence is increasing [13–15]. Heat stress, as defined by the American Conference of Governmental Industrial Hygienists [14,16], is the amount of heat load or net heat to which a worker is subjected [17]. Work rate, clothes, ambient temperature, and humidity influence heat load. Moreover, indoor climates in non-air-conditioned buildings are likely to increase the risk of heat waves, especially in low-income countries as climate change and energy crises, and it can cause heat stress to residents and workers who spend most of their time indoors [18,19]. High workplace heat load can have a variety of effects on a person’s metabolism, body temperature, heart rate, and blood pressure. However, it will ultimately lead to illness and an increase in work errors [20]. Excessive heat exposure has been linked to consequences such as heat exhaustion, neurological and psychiatric diseases, and decreased labor productivity [21].

Inflammation is a natural and necessary response to injury or infection, where the body’s immune system activates to protect itself and promote healing. However, long-term chronic inflammation is a sustained immune response lasting months or years arising from factors such as persistent infections, exposure to irritants, and unresolved inflammatory responses that contribute to its development. The long-term presence of chronic inflammation can lead to cardiovascular disease, autoimmune disorders, and cancer [22,23]. Various blood and tissue factors including cytokines interleukin-1beta (IL-1β), interleukin-6 (IL-6), and Tumor Necrosis Factor-Alpha (TNF-α), several serum proteins such as C-reactive protein (CRP), intercellular cell adhesion molecules (ICAM) and vascular cell adhesion molecules (VCAM), which are considered as inflammatory agents, are altered during inflammation and provoke exacerbation [22]. Cytokines constitute a group of proteins or soluble glycoproteins that transmit vital signals between immune and non-immune cells. They also possess pro-inflammatory, anti-inflammatory, and and immunosuppressive activities [23]. Studies have shown that IL-1, IL-6, TNF-α, and CRP play a major role in the pathogenesis of heat-related illnesses such as heatstroke, and their serum levels are greatly increased by heat [11,24,25].

There is evidence of a positive correlation between heat stroke patients’ body temperature and IL-1β levels [26]. TNF-α production also occurs with thermal exposure [25]. When the body temperature rises, certain tissue-resident immune cells, such as macrophages, produce proinflammatory cytokines such as IL-1β, IL-6, and TNF-α [25,27]. Evaluations in a mouse model have indicated that heat stress causes an increase in inflammatory cytokines, reactive oxygen species (ROS), and nitric oxide (NO) by activating nuclear factor kB (NF-kB) as the master regulator of inflammatory responses [28,29], and IL-6 and IL-1 mediate the release of acute-phase proteins such as CRP, from liver cells. In line with this data, research has revealed that individuals who report job-related stress often exhibit elevated CRP levels, which are associated with atherosclerosis [30]. Furthermore, male cyclists who trained in hot conditions and had limited fluid intake experienced an increase in IL-6, cortisol, and CRP [31].

To evaluate workplace thermal conditions and the associated heat stress on people, many thermal indicators have been presented and used over many years [32,33]. One of the most widely used and standard indicators in this area is the wet-bulb globe temperature (WBGT). This index was introduced in 1957 by Yaglou [34] and approved in the ISO7243:1989 standard [35]. Despite some criticism, the WBGT is the most common measure of heat stress as the calculation is formulated to accommodate human heat exchanges with the environment and the influence of work according to temperature and humidity [14,32]. Ultimately, calculations for potential heat stress involve calculations of the WBGT index alongside published threshold limit values (TLV).

In this study, the population of interest to explore the impact of heat stress on inflammatory markers was traditional artisan bakers in Iran who work indoors in front of hot ovens. Studies show that WBGT indices are high in people who work long hours in front of artisan bakery furnaces and ovens [2,36]. This work is considered to be moderately strenuous. The strenuousness of a job impacts on the TLV. That is, work that involves greater strenuousness will have lower TLV for managing heat stress.

Iranian bakers start work at 4 a.m. and 3 p.m. and generally work two shifts per day, six days a week. It takes about 5 hours overall to prepare the dough to bake the bread. There are four distinct job roles undertaken by traditional bakers in the course of making bread. The initial preparation is undertaken by a dough-maker who mixes flour and water to make a dough of a spongy consistency. Next, dough-formers knead, portion and shape the dough. Then the dough-baker puts the cut dough into the open ovens and manages the heat until the dough is baked and becomes bread. Finally, a bread-taker retrieves the freshly baked bread and put it on a counter ready to be sold. The risk for heat stress may differ according to role. For moderately strenuous jobs a WBGT TLV of 28°C or lower should be sought to reduce the risk of heat stress and its consequences [2].

An investigation of the effects of high heat on broilers found that high heat increased inflammation and the release of IL-1 and IL-6 [36]. Considering the effects of high heat on humans, under carefully controlled experimental conditions, Kaldur *et al*. evaluated the effect of indirect heat on vascular stiffness, oxidative stress, and inflammation in healthy, physically active young men [37]. They found that exposure to high temperature (42°C) and a relative humidity of 18% in a climatic chamber during a 10-day heat and exercise study protocol, yielded a significant relationship between heat rise and hardening of arterial blood vessels [38]. Based on the findings, they also asserted there is a correlation between high heat and CRP and IL-6 levels seen in the study participants. This led to increased CRP and IL-6 levels significantly [38,39]. Since heat propounds inflammation, and inflammatory factors underlie the onset of many disorders including autoimmune disease [40], cancers [41], organ failure [42], and psychological consequences like depression [43], we determined a need to study the level of the inflammation criteria IL-1, IL-6 and, TNF-α and CRP in the serum of workers who are exposed to a high heat environment during their work. This study investigated how excessive heat in working environments affects people’s inflammatory conditions. It also recommended some actions to alleviate hazardous conditions for workers.

## Materials and methods

### Design and participants

This is a case-control study of men who were referred to the specialized Center of Occupational Medicine in Shahroud for their annual health assessment in 2019–2020. Using information from serum samples, the participants were divided into two groups: case (n = 47) and control (n = 58). The normal range of IL-6, IL-β, TNF-α, and CRP are 2–60 ng/L, 20-60pg/L, 3- 90ng/L and 3–10 mg/L respectively. Thus, the case group (abnormal inflammation) included people with a higher-than-normal range of one or more of the four inflammatory factors IL-6, IL-1β, TNF-a, and CRP, and participants with serum samples in the normal range for these four inflammatory factors became the control group (normal inflammation).

96 men bakers working in artisan bakeries performing one of four jobs and 15 administrative officers, who all met the study criteria and filled out the informed consent form, were recruited. Six participants subsequently withdrew due to infection yielding a final sample of 105 participants.

### Ethical considerations

This project was approved by the Research Ethics Committee of Shahroud University of Medical Sciences under No. IR.SHMU.REC.1397.211. The study was conducted in 2020, according to the principles of the Declaration of Helsinki.

The details of the project were explained to all participants in a transparent manner and the personal information of the individuals was kept strictly confidential. All participants entered the study voluntarily after giving informed written consent.

### Sample size

Sufficiency of sample size was based on the mean and standard deviation of serum level of TNF-α in heat stroke patients in Chang’s study of the role of cytokines in heat stroke. Considering a significance level of 0.05 and statistical power of 80%, the minimum sample size required for each group needed to reach 25 considering a 10% loss to our samples. Also, ratio of control to case assumed 1. Finally, in order to increase the study power, the research group doubled the sample size in each group and the sample size was increased to 50 people in each group. That is, the planned minimum sample size was increased to 100.


$$n_{1}=n_{2}=\frac{{\left(Z_{1-\frac{\alpha }{2}}+Z_{1-\beta }\right)}^{2}(\delta_{1}^{2}+\delta_{2}^{2})}{{\left(\mu_{1}-\mu_{2}\right)}^{2}}$$

For all participants: inclusion criteria were full-time employment for at least one year, and men aged 18 to 60 years old; exclusion criteria included suffering from diseases such as high blood pressure, heart disease, and fever.

### Data collection

To conduct this study, the purpose of the study and the conditions for collecting samples through oral explanations were described for all individuals. After declaring consent to participate in the study, participants filled out a written informed consent form. Subsequently, among all individuals who entered the study after completing the written consent form, 6 individuals were excluded due to infection. The study was then carried out on the remaining participants. A questionnaire was used to collect demographic and personal information, including age, smoking status, vegetable consumption, regular exercise, work experience, daily working hours, and job type. Iranian bakeries start at 4 a.m. and 3 p.m. in two shifts per day. It takes about 5 hours to prepare the dough to bake the bread. Participants’ blood samples (5ml) were collected from a cubital vein by a laboratory technician skilled in phlebotomy in the morning, before bakers’ working hours ended. After that, samples were left at room temperature for 15–30 minutes for clotting. After removing the clot by centrifuging 1500g for 10 minutes at 4°C, serum specimens were collected and kept at -70°C until analysis.

In this study various variables were investigated as follows: BMI(Kg/m^2^) was calculated using height (m) and weight (kg), age (years), work experience (years), and exposure to heat using a dosimeter were recorded quantitatively. Participants were categorized based on BMI (>25 or ≤25), heat exposure (≤28°C or >28°C), and daily work hours (≥8 hours or <8 hours). Additional qualitative data on exercise, smoking, vegetable consumption, and job type were collected through interviews. The first three were categorized as "Yes" or "No" and the occupation type was classified as "official" or "baker."

The wet bulb globe temperature (WBGT) index was used to evaluate heat stress in bakery workers. Previous studies have confirmed an acceptable association of the WBGT index with physiological indicators of the body such as core temperature [44–46]. To take measurements, we used a digital WBGT meter made by Casella, Australia (MK427JY model) which is designed to monitor the risk for heat stress in employees working in hot environments. Because of environmental inconsistency, and according to the standard protocol, measurements were made at three heights: head (1.7 m), trunk (1.1 m) and ankles (0.1 m) and their average were determined (Eq 1) [47]. Also, based on the measurement of temperature values at different times during work shifts (both at rest and during work), the WBGT index was calculated using the mean weighted-time relation (Eq 2) [48].


$$WBGTi=\frac{WBGT_{\mathrm{head}}+(2\times WBGT_{\mathrm{abdomen}})+WBGT_{\mathrm{leg}}}{4}$$ (Eq 1)

$$WBGT_{\mathrm{average}}=\frac{\sum_{i=1}^{n}\left(ti\times WBGTi\right)}{\sum_{i=1}^{n}\;ti}$$ (Eq 2)

To determine the permissible values of the WBGT index of the environment, the working metabolism of individuals was determined using ISO-8996 Standard tables to judge the level of heat stress. Using this approach, and accounting for the postures, types and amounts of each individual’s activity, the metabolism of bakery workers was calculated as 200–350 kcal/h, which is in the average work range. According to this Standard, 8 hours working time corresponded to the pattern of 75–100% of baker’s working time and the acceptable limit of WBGT index was 28°C.

### Evaluation of serum inflammatory factors

Serum concentration of inflammatory factors IL-1β, IL-6, TNF-α, and CRP were determined with an enzyme-linked immunoassay (ELISA) method using the Bioassay Technology Laboratory kits. As previously described, a 5 ml blood sample was collected from the right antecubital vein of all participants in the morning, while they were seated. The blood samples were centrifuged for 20 minutes at 1500g and serum were separated and collected in 0.5 ml microtubes and were then refrigerated at -70°C until analysis. All tests were performed in the central laboratory of Shahroud University of Medical Science.

### Statistical analyses

Kolmogorov-Smirnov tests were used to evaluate the normality of data distribution. Descriptive data were reported using mean, standard deviation, frequency and frequency percentage. Then, *χ*2 analyses was used to check the relationship of qualitative variables with study groups and independent t-test analyses was used to check the relationship of quantitative variables with study groups.

Adjusted odds ratio and their 95% confident interval were calculated by including all variables in the multivariate model to control for confounding effects. All statistical calculations were performed with SPSS version 26 (SPSS Inc, Chicago, Ill, USA) and p-value < 0.05 was considered statistically significant. Graphs were drawn using GraphPad Prism version 9 software.

## Results

### Descriptive information

The 105 subjects were all male and had a mean age of 35.67±8.30 years. Body mass index (BMI) was 25.24±4.63 kg/m^2^, work experience 13.56±9.01 years, and daily working hours 9.67 ± 1.42 hours. Table 1 shows the frequencies of qualitative and quantitative demographic variables for case and control groups, and the validity of qualitative variables as well as the normality of quantitative data has been reported by examining the results of Chi-square and Kolmogorov-Smirnov tests, respectively.

**Table 1:** Demographic information and other characteristics in cases and control groups.

Parameter	Control	Case	OR	95%CI			P-value		
**Age (years)** *	34.23±7.40	36.84±8.85	1.04	0.99	1.09			0.11	
**Experience (years)** *	12.93±7.55	14.06±10.08	1.01	1.00	1.06			0.52	
**BMI (kg /m^2^)** **									
≤25	24 (51.1%)	32 (55.2%)	Reference	Ref	Ref			Ref	
>25	23 (48.9%)	26 (44.8%)	0.85	0.39	1.83			0.67	
**Vegetable Consumption** **									
yes	39 (83.0%)	47 (81.0%)	Reference	Ref	Ref			Ref	
no	8 (17.0%)	11 (19.0%)	0.88	0.32	2.39			0.80	
**Smoking** **									
Yes	30 (63.8%)	32 (55.2%)	1.43	0.65	3.15			0.38	
No	17 (36.2%)	26 (44.8%)	Reference	Ref	Ref			Ref	
**Daily work hours** **									
<8	16 (8.3%)	12 (16.7%)	Reference	Ref	Ref			Ref	
>8	31 (21.4%)	46 (53.6%)	1.98	0.82	4.75			0.13	
Exercise**									
yes	6 (12.8%)	16 (27.6%)	Reference	Ref	Ref			Ref	
no	41 (87.2%)	42 (72.4%)	2.60	0.93	7.31			0.06	
**Job** **									
**Official**	4 (3.6%)	11 (19%)	Reference	Ref	Ref			Ref	
**Bread-taker**	5 (10.6%)	16 (27.6%)	1.16	0.25	5.33			0.84	
**Dough-making**	6 (12.8%)	8 (13.8%)	0.48	0.10	2.30			0.36	
**Dough-forming**	25 (53.2%)	19 (32.8%)	0.27	0.76	1.00			0.05	
**Baker**	7 (14.9%)	4 (6.9%)	0.20	0.39	1.11			0.06	
Testate of Exposure to thermal stress**								
Acceptable limit	21 (44.7%)	17 (29.3%)	Reference	Ref	Ref			Ref	
Unacceptable limit^1^	26 (55.3%)	41 (70.7%)	1.95	0.87	4.36			0.10	

*Independent T-test

**Chi Square Test, OR by Binary Logistic Regression.

^1^ Exceeds the workplace standard for heat stress.

### Laboratory findings

**Fig 1:**
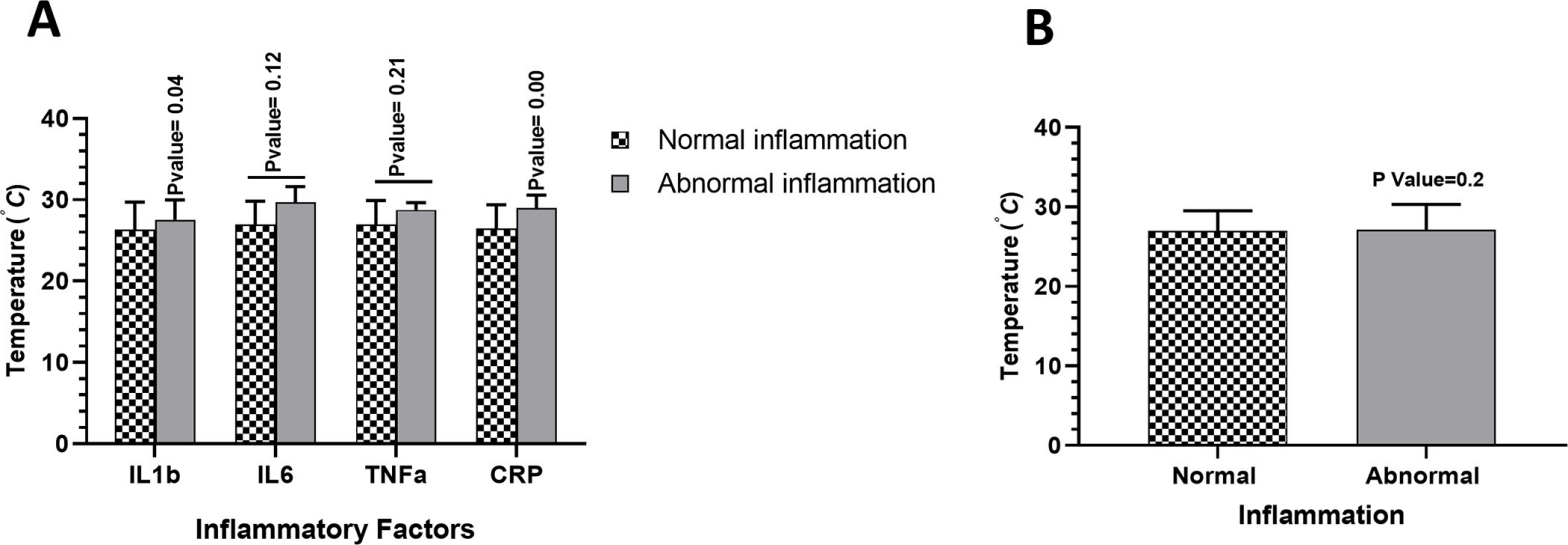
Investigating the effect of increasing temperature on inflammatory status in both normal (dotted columns) and abnormal (grey columns) inflammation groups. **(A)** Increasing the temperature beyond the acceptable limit WBGT (28֯C) leads to an increase in any of the four inflammatory factors. **(B)** Increasing temperature effect on total inflammation (including all 4 inflammatory factors: IL1β, IL6, TNFα and CRP) in participants with normal/abnormal inflammation.

Serum levels of IL-1β, IL-6, TNF-α, and CRP in the case and control groups were evaluated using ELISA. There were higher levels of all four factors among heat-stressed participants, but independent t-test analyses showed that only the augmentation of IL-1β (p-value = 0.03, 95% CI of diff = -2.85 to -0.085) and CRP (p-value < 0.01, 95% CI of diff = -4.11 to -1.0) was statistically significant. Following the results, we divided the sample into two categories: case (people with abnormal inflammation) and control (people with normal inflammation), and evaluated their responses to increasing temperature. The findings revealed that, as the temperature increased, there was an augmentation in abnormal inflammation in the case group; however, this effect was not significant (See Fig 1).

### Multivariate logistic regression model for total inflammation

To further understand the significantly raised occupational heat stress levels of IL-1β and CRP, a multivariate model based on the enter method was used. The analysis included all variables and adjusted odds ratios and their 95% confidence intervals were used, as presented in Table 2.

**Table 2:** Multivariate logistic regression model of risk factor for total inflammation*.

Parameter	OR	95% CI		p-value
		Lower	Upper	
**Age (Years)**	1.04	0.94	1.14	0.47
**BMI (kg /m2)**	1.01	0.91	1.12	0.81
**Experience (years)**	0.98	0.90	1.07	0.74
**Testate Exposure to thermal stress**				
Acceptable limit	Ref	Ref	Ref	Ref
Unacceptable limit^1^	4.11	1.25	13.51	0.02
**Job**				
Official	Ref	Ref	Ref	Ref
Bread-taker	0.49	0.07	3.63	0.48
Dough-making	0.17	0.21	1.35	0.09
Dough-forming	0.13	0.21	0.77	0.02
Baker	0.08	0.01	0.71	0.02
**Vegetable Consumption**				
Yes	Ref	Ref	Ref	Ref
No	0.38	0.08	1.87	0.23
**Smoking**				
No	Ref	Ref	Ref	Ref
Yes	1.13	0.41	3.09	0.81
**Exercise**				
Yes	Ref	Ref	Ref	Ref
No	0.23	0.05	1.04	0.06
**Daily work hours**				
<8	Ref	Ref	Ref	Ref
>8	1.08	0.26	4.44	0.91

* Total inflammation includes two inflammatory factors, CRP and IL1β, that showed significant changes in studied groups after heat exposure, as depicted in Fig 1A.

^1^ Exceeds the workplace standard for heat stress.

The effect estimates for heat exposure varied considerably between bivariate (OR: 1.948, 95%CI: 0.870 4.363) in Table 1 and multivariate analysis (OR: 4.114, 95% CI 1.254, 13.514) in Table 2. the analysis was repeated two more times. Once by removing variables one by one and the second time by adding variables one by one. In both models, the job variable has the greatest impact on etestate exposure to thermal stress.

So that by removing the job variable from the model, the adjusted OR of heat exposure decreases to 2.25 (95%CI 0.928, 5.458), which means that the heat exposure variable is no longer statistically significant (p = 0.073). However, due to the high importance of the job variable in our study and its determining role in the heat exposure of the participants, we cannot remove job variable from the final model. The multivariate model without the job is shown in supplementary material (S1 File, Table 1).

If in the sub-groups of the job variable, the official occupation is removed and only baker types are included in the multivariate analysis, the adjusted odds ratio changes to 4.844 (95%CI 1.380, 16.999), which is statistically significant (p = 0.014) (S1 File, Table 2).

The multivariate regression model found that exposure to thermal stress, and two of the bakers’ job type (dough forming and baker) affected total inflammation. As shown in Table 2, participants exposed to high temperatures and above the TLV WBGT have a 4.11-fold increased probability of having total inflammation. Specifically, two types of bakery jobs–dough-forming and baker–have an influence on the total inflammation by 1.2-fold and 0.08-fold, respectively. Age, BMI, experience, vegetable consumption, smoking, exercise, and daily work hours despite having an effect on total inflammation, were not significant.

## Discussion

The objective of this study was to investigate the effects of prolonged exposure to ambient heat in specific occupations on inflammatory changes in individuals employed in those fields. Initially, all participants (bakers and employees) were categorized into two groups—normal and abnormal—according to their inflammatory status. Subsequently, their demographic status, exposure to environmental heat at work, and the ensuing effects on inflammation were evaluated. Investigation of serum concentrations of IL-1β, IL-6, TNF-α, and CRP as indicators of systemic inflammation in individuals revealed that the increase of IL-1β and CRP in bakers was significant. Although IL-6 and TNF-α were also increased in workers with an excessively high WBGT index, these increases were not statistically significant. While this result was not in line with expectations, it was not, in fact, at odds with some studies in the literature. For example, Wright *et al*. [49] designed a study that examined the effect of exercise in a high temperature / humid environment that represented occupational heat stress. They hypothesized that age would make men more susceptible to heat illnesses, as they had higher circulating TNF-α and IL-6 levels at baseline. Whilst ’working conditions’ (for example exercise) raised circulating TNF-α and IL-6 levels in both older and younger groups of men in their experiment, the changes observed were similar. That is, just as in this study, environmental conditions elevated TNF-α and IL-6 measures to levels indicative of persistent low-grade inflammation, but the difference between the two groups was not substantial enough to be statistically significant.

For a more comprehensive analysis of the participants’ inflammatory condition, they were divided into case and control groups based on their abnormal/normal levels of four inflammation factors. Their inflammatory condition was evaluated following exposure to temperatures exceeding the WBGT index. Univariate regression analyses revealed no significant difference between total inflammation and exposure to high environmental heat. Nevertheless, when key variables were entered into the multivariate logistic regression model, a four-fold increase in total inflammation was observed due to exposure to high heat. A non-significant result of IL-6 and TNF-α can be attributed to the overall result of inflammation in the univariate analysis. However, in the multivariate logistic regression model, the effect of excessive temperature on total inflammation increased when different factors interacted with each other, and confounding factors were controlled. The analyses also indicated a relationship between the type of bakery task and the total inflammation. Among the bakery job roles, bakers who do bread baking had the most direct contact with direct heat and worked in the hottest place for most of their working day. After that, dough-formers, bread-takers, and dough-makers were exposed to more heat and high ambient temperatures. As a result of the multivariate logistic regression test, bread bakers and dough-formers were found to have the highest likelihood of significantly increasing total inflammation, whereas dough makers and bread takers showed no significant correlation with total inflammation when compared to officials. This significant correlation confirmed the hypothesis of the probability effect of high WBGT on the increase in total inflammation in workers.

The results demonstrate that workers can be negatively affected by high-heat conditions. Although there may be a practical barrier to installing more ventilation in hot indoor environments, the results suggest that less specialism in the working day would benefit traditional bakers. It is recommended that bakers operate a rotating work schedule as dough-maker, dough-former, baker, and bread-taker to minimize exposure to excessive heat. At hot temperatures, water consumption plays a critical role in controlling inflammation. Bakers can prevent dehydration by drinking copious amounts of liquid other than alcohol and caffeine. In fact, an examination of the effects of acute exercise in the heat on biomarkers of stress and inflammation found that IL-6 and cortisol increased only when fluid intake was restricted [31].

Ultimately, although substantial strides have been taken to ameliorate work-related health hazards, the notion of a risk-free work environment is utopian as a fundamental aspect of many occupational and environmental risk factors is that they are integral to the job. Bakers need heat to make bread. No heat, no job. Understanding risks, incorporating appropriate and sufficient surveillance, and mitigating actions are critical to the health of employees (and businesses). We have provided indicative actions in the previous paragraph. As noted by Chirico & Magnavita [49], risk assessment is necessary, but not sufficient in managing occupational heat stress. That is, health surveillance is also a critical part of employee health. Critically, there is some need for a personalized perspective in understanding an employee’s health risk because individual differences dictate non-work risks for health problems which can interact with work risks [19]. Thus, if high heat stress cannot be prevented, health surveillance should be a part of working conditions. In addition, health surveillance should be a part of general strategies to ameliorate workplace risk because of high environmental heat. Thus, if high heat stress cannot be reduced, then health surveillance should be a part of the working conditions, as well as the general strategies to ameliorate the workplace risk because of the high environmental heat [50].

Despite the importance of this topic, there are not enough comprehensive studies investigating the effect of occupational conditions such as heat, on physiological and clinical changes in workers [50,51]. ISO 12894 does not explicitly call for individual tests of heat tolerance to be conducted in the section considering “Individual heat strain monitoring [52], but equally this international standard does require those overseeing the welfare of those working in conditions in which exposure to high heat cannot be avoided. In our study, we tried to consider various influential factors to health outcomes, including markers of nutrition, exercise and smoking habits, BMI, and working hours. In future studies, however, it is necessary to carefully examine the factors related to exposure. These factors include the intensity of the heat encountered, the number of working days per week, and the type of thermal source encountered, among others. These are variables we would expect to be part of an annual health assessment for those working in high-heat-stress conditions. Responses should indicate those whose health should be closely monitored.

The all-male sample was a limitation, though this is typical of Iran’s occupation. According to a recent study conducted in traditional bakeries in Lebanon, women were more likely to experience heat-stress-related symptoms [50]. This should be confirmed in future studies. Although this study focused on inflammatory factors in workers’ plasma, it may have been beneficial to include other inflammatory factors related to the heart, liver, and kidney. These factors include D-Dimer, liver enzymes, and a-keratin. Similarly, molecular investigations could provide affirmative evidence for plasma cytokine changes. While our study’s methodology was accurate, limitations consisted of small sample size and lack of diversity in the jobs examined. A larger sample size from different regions of the country with different climates among a large population of heat-exposed workers in future studies would strengthen our findings. Moreover, future studies should incorporate participants’ drinking water as a crucial criterion for controlling body temperature.

The novel aspect of our study lies in examining long-term exposure to ambient heat in specific occupations and its effect on inflammation changes in individuals engaged in those occupations. In this way, the participants were initially divided into two groups, normal and abnormal, based on their inflammatory status. After that, the environmental thermal effect of jobs was evaluated on these people’s inflammatory status.

## Conclusions

This study affirmed that prolonged exposure to unregulated occupational environmental stress such as heat, heightens risk factors such as inflammation in workers. Consequently, establishing standardized working conditions in businesses becomes crucial for maintaining workers’ health.

## Supporting information

S1 Data Raw data file.xlsx and.sav format of raw data of the study that is available by Excel and SPSS software. (XLSX)

S1 File Supplementary. The multivariate models in bakers after excluding officials.
(ZIP)

S2 File Questionnaire. The original language questionary form to collect data from participant.
(DOCX)

S3 File Multivariete report. The output file of the data analysis that contains the EXP(B) report.
(DOCX)
