# Supplementary material for: The influence of occupational heat stress on serum inflammatory cytokines among traditional bakery workers in Iran
Source: PLoS One. 2024 May 6;19(5):e0302847. doi: 10.1371/journal.pone.0302847 (PMC11073666; doi:10.1371/journal.pone.0302847)
Supplement: S2 File — The original language questionary form to collect data from participant. (DOCX) [file pone.0302847.s003.docx]

**Questionnaire (original language)**

1. **نوع پخت نان:** 1)لواش☐ 2)تافتون ☐ 3)سنگگ☐ 4)بربری☐
2. **شغل فعلی** شما (نوع وظیفه) چیست؟

1)کارگر خمیر گیر و چانه گیر☐ 2)چونه پهن کن یا وردنه زن☐ 3)شاطر☐ 4)نان درآر☐

1. **سن (سال):** ............................. 4- **قد** **(سانتی­متر):** ............................ 5- **وزن (کیلوگرم):** ..............................
2. **وضعيت تأهل:** 1- مجرد ☐ 2- متأهل ☐
3. **تعداد افراد تحت تکفل**: ................................
4. **سابقه کاردر شغل نانوایی**: ................................. سال
5. **کل سابق کار شما(در کل دوران کاری)**: ................................. سال
6. **میزان تحصیلات:** 1- زیر دیپلم ☐ 2- دیپلم ☐ 3- فوق دیپلم و بالاتر ☐
7. **آیا شما دارای شغل دوم هستید**؟ 1- بلی ☐ 2- خیر ☐
8. **به طور متوسط، چند ساعت در روز کار می کنید** ؟ ...............................
9. **آیا شما در طول هفته بصورت منظم ورزش می کنید(حداقل سه بار در هفته)؟** 1- بله ☐ 2-خیر ☐
10. **آیا در هفته حداقل دو مرتبه میوه جات مصرف می کنید ؟**

1.بله 2. خیر

1. **آیا در هفته حداقل دو مرتبه سبزی جات مصرف می کنید ؟**

1.بله 2. خیر

1. **آیا در شبکه های اجتماعی موبایلی(تلگرام،لاین،اینستاگرام و...) فعالیت گسترده دارید(حداقل یک ساعت در روز)** 1.بله 2. خیر
2. **آیا شما سيگار می کشيد؟** 1- بله ☐ 2- خير ☐
3. **تعداد نخ مصرف سیگار در شبانه روز : .**
