# Supplementary material for: The influence of occupational heat stress on serum inflammatory cytokines among traditional bakery workers in Iran
Source: PLoS One. 2024 May 6;19(5):e0302847. doi: 10.1371/journal.pone.0302847 (PMC11073666; doi:10.1371/journal.pone.0302847)
Supplement: S3 File — The output file of the data analysis that contains the EXP(B) report. (DOCX) [file pone.0302847.s004.docx]

**Table 1. Multivariate logistic regression model of risk factor for total inflammation without job**

| **Parameter** | | **OR** | | **95% CI** | |  | | **P-value** | |
| --- | --- | --- | --- | --- | --- | --- | --- | --- | --- |
|  |  |  |  | **Lower** | | **Upper** | |  |  |
| **Age (Years)** | | 1.08 | | 0.10 | | 1.18 | | 0.06 | |
| **BMI (kg /m2)** | | 1.01 | | 0.92 | | 1.11 | | 0.82 | |
| **Experience (years)** | | 0.95 | | 0.88 | | 1.03 | | 0.19 | |
| **Vegetable Consumption** | |  | |  | |  | |  | |
| Yes | | Ref | | Ref | | Ref | | Ref | |
| No | | 0.56 | | 0.14 | | 2.30 | | 0.42 | |
| **Smoking** | |  | |  | |  | |  | |
| No | | Ref | | Ref | | Ref | | Ref | |
| Yes | | 1.21 | | 0.48 | | 3.05 | | 0.69 | |
| **Daily work hours** | |  | |  | |  | |  | |
| <8 | | Ref | | Ref | | Ref | | Ref | |
| >8 | | 1.64 | | 0.63 | | 4.30 | | 0.31 | |
| **Exercise** | |  | |  | |  | |  | |
| Yes | | Ref | | Ref | | Ref | | Ref | |
| No | | 0.26 | | 0.07 | | 1.01 | | 0.05 | |
| **Testate Exposure** **to thermal stress** | |  | |  | |  | |  | |
| Acceptable limit | | Ref | | Ref | | Ref | | Ref | |
| Unacceptable limit | | 2.25 | | 0.93 | | 5.46 | | 0.07 | |

**Table 2. Multivariate logistic regression model of risk factor for total inflammation in bakers**

| **Parameter** | **OR** | **95% CI** | | **P-value** |
| --- | --- | --- | --- | --- |
|  |  | **Lower** | **Upper** |  |
| **Age (Years)** | 1.04 | 0.94 | 1.16 | 0.41 |
| **BMI (kg /m2)** | 1.01 | 0.91 | 1.12 | 0.90 |
| **Experience (years)** | 0.97 | 0.89 | 1.07 | 0.59 |
| **Vegetable Consumption** |  |  |  |  |
| Yes | Ref | Ref | Ref | Ref |
| No | 0.19 | 0.02 | 1.80 | 0.14 |
| **Smoking** |  |  |  |  |
| No | Ref | Ref | Ref | Ref |
| Yes | 1.48 | 0.48 | 4.53 | 0.49 |
| **Daily work hours** |  |  |  |  |
| <8 | Ref | Ref | Ref | Ref |
| >8 | 1.38 | 0.41 | 4.58 | 0.60 |
| **Exercise** |  |  |  |  |
| Yes | Ref | Ref | Ref | Ref |
| No | 0.12 | 0.01 | 0.96 | 0.05 |
| **Testate Exposure** **to thermal stress** |  |  |  |  |
| Acceptable limit | Ref | Ref | Ref | Ref |
| Unacceptable limit | 4.84 | 1.38 | 17.00 | 0.01 |
| **Job** |  |  |  |  |
| Bread-taker | Ref | Ref | Ref | Ref |
| Dough-making | 0.35 | 0.06 | 1.93 | 0.23 |
| Dough-forming | 0.25 | 0.07 | 0.93 | 0.04 |
| Baker | 0.16 | 0.02 | 1.03 | 0.05 |
